# A Relationship-Oriented Model of Research Participation: The Brain Health Community Registry

**DOI:** 10.1093/geroni/igab046.2376

**Published:** 2021-12-17

**Authors:** Andrea Gilmore Bykovskyi, Haley Fuhr, Shannon Mullen, Laura Block, Clark Benson, Danielle Harris, Quinton Cotton, Jessica Kendall

**Affiliations:** 1 University of Wisconsin-Madison, Madison, Wisconsin, United States; 2 University of Wisconsin-Madison, University of Wisconsin-Madison, Wisconsin, United States; 3 University of Wisconsin - Medicine & Public Health, Fitchburg, Wisconsin, United States

## Abstract

Historically excluded and minoritized populations are significantly under-included in health studies of Alzheimer’s disease and related dementias (ADRD) despite bearing a disproportionate burden of disease—evidenced by higher incidence, prevalence, and poorer health outcomes. Meaningful progress toward identifying and alleviating causes of health disparities in ADRD necessitate effective and scalable approaches for broadening inclusion in research. Rigorous studies evaluating research participation among minoritized populations are limited and have predominantly focused on individual-level factors and behavioral change (i.e. religiosity, willingness). These approaches frequently overlook the influence of unequally distributed structural and social determinants on participation despite the compounded financial, social, emotional, and logistical consequences that result from ADRD. Using an intersectional and social justice lens, we developed the Participant and Relationship-Oriented Research Engagement Model, which characterizes research as a form of relationship and extends social determinants frameworks to the context of research participation. We report core components of the model and its application in the design and preliminary evaluation of the Brain Health Community (BHC) Registry, which features proactive and systematic evaluation of potential unmet needs among prospective participants, and connections to relevant services (i.e. respite care, adaptive devices). Preliminary testing of the model and participant feedback on the BHC suggest it is a feasible approach to research engagement, and that associated assessment tools and resource protocols are acceptable and sufficiently adaptable to heterogeneous sets of unmet needs. Primary challenges include ongoing assessment of engagement and routine changes in service ability, which can be addressed through community-based resource networks.

